# Clinical procedure for colon carcinoma tissue sampling directly affects the cancer marker-capacity of VEGF family members

**DOI:** 10.1186/1471-2407-12-515

**Published:** 2012-11-13

**Authors:** Sarah Pringels, Nancy Van Damme, Bram De Craene, Piet Pattyn, Wim Ceelen, Marc Peeters, Johan Grooten

**Affiliations:** 1 Department of Biomedical Molecular Biology, Ghent University, Technologiepark 927, Zwijnaarde, 9052, Belgium; 2 Department of Surgery, Ghent University Hospital, De Pintelaan 185, Gent, 9000, Belgium; 3 Department for Molecular Biomedical Research VIB, Zwijnaarde, 9052, Belgium; 4 Department of Oncology, Antwerp University Hospital, Wilrijkstraat 10, Edegem, 2650, Belgium

**Keywords:** VEGF family members, Colon cancer, Sampling procedure, Biomarker, Hypoxic stress

## Abstract

**Background:**

mRNA levels of members of the Vascular Endothelial Growth Factor family (VEGF-A, -B, -C, -D, Placental Growth Factor/PlGF) have been investigated as tissue-based markers of colon cancer. These studies, which used specimens obtained by surgical resection or colonoscopic biopsy, yielded contradictory results. We studied the effect of the sampling method on the marker accuracy of VEGF family members.

**Methods:**

Comparative RT-qPCR analysis was performed on healthy colon and colon carcinoma samples obtained by biopsy (n = 38) or resection (n = 39) to measure mRNA expression levels of individual VEGF family members. mRNA levels of genes encoding the eicosanoid enzymes cyclooxygenase 2 (COX2) and 5-lipoxygenase (5-LOX) and of genes encoding the hypoxia markers glucose transporter 1 (GLUT-1) and carbonic anhydrase IX (CAIX) were included as markers for cellular stress and hypoxia.

**Results:**

Expression levels of *COX2*, *5-LOX, GLUT-1* and *CAIX* revealed the occurrence in healthy colon resection samples of hypoxic cellular stress and a concurrent increment of basal expression levels of VEGF family members. This increment abolished differential expression of *VEGF-B* and *VEGF-C* in matched carcinoma resection samples and created a surgery-induced underexpression of *VEGF-D*. *VEGF-A* and *PlGF* showed strong overexpression in carcinoma samples regardless of the sampling method.

**Conclusions:**

Sampling-induced hypoxia in resection samples but not in biopsy samples affects the marker-reliability of VEGF family members. Therefore, biopsy samples provide a more accurate report on VEGF family mRNA levels. Furthermore, this limited expression analysis proposes VEGF-A and PlGF as reliable, sampling procedure insensitive mRNA-markers for molecular diagnosis of colon cancer.

## Background

Colorectal cancer is the second most commonly diagnosed cancer in females and the third in males. It is the second leading cause of cancer-related death [1]. Worldwide, it accounts for over 1.2 million new cases every year, and in 2008 it caused about 608,700 deaths. Colon carcinoma evolves from a premalignant adenoma precursor stage or polyp. The progression from adenoma to carcinoma is a multistep process involving cumulative genetic and epigenetic alterations in proto-oncogenes, tumor suppressor genes and DNA repair genes [2-4].

Colon carcinoma tissue samples have been intensively studied in search for tissue-based diagnostic, prognostic and predictive markers. Samples are routinely obtained by two different clinical procedures. During colonoscopy, which is the gold standard for detection of colon carcinoma and adenoma, biopsies of polyp-like extrusions are obtained for pathological examination, and these extrusions are removed whenever possible. In surgical resection, carcinoma-like outgrowths are removed by cutting out part of the colon containing the suspected outgrowth as well as some surrounding healthy tissue. However, little is known about the impact of the sampling method on the overall condition of the sampled tissue or the expression levels of potential cancer biomarker genes.

Vascular Endothelial Growth Factor (VEGF; VEGF-A) has long been proposed as a biomarker for cancer as well as a target for anti-angiogenic cancer therapy. Several studies consistently showed elevated VEGF-A expression levels in most solid tumors, including colon carcinoma [5-9]. Furthermore, these elevated expression levels have been correlated with tumor progression [10-12]. VEGF-A is an inflammation and hypoxia responsive gene, and its biomarker function is believed to be related to the hypoxic growth conditions characteristically associated with rapidly growing solid tumors and to its ability to promote the development of new vasculature [11,13].

Fewer studies addressed the mRNA expression levels in colon cancer of the other VEGF family members: VEGF-B, VEGF-C, VEGF-D and Placental Growth Factor (PlGF). Furthermore, some of these studies reported contradictory results. As such, similar expression levels of *VEGF-C* in healthy and carcinoma tissue were reported in three studies [5,8,14]. However, other studies reported higher levels [6,7] that were correlated with lymph node metastasis and poor prognosis [8].

We believe that some of these controversial findings might have resulted from the use of different types of colon tissue samples. Several studies performed expression analysis on samples obtained during surgical resection [5,7,8]. Others used biopsies obtained during colonoscopy [14] or did not specify the sampling method [6]. Yet, both sampling procedures differ strikingly; the acquirement of colon biopsies requires only minutes, whereas during surgical resection part of the colon is clamped off for a considerable length of time. To examine to what extent the sampling procedure may affect VEGF gene expression, we analyzed mRNA expression levels of all five VEGF family members in colon carcinoma samples obtained by biopsy and in others obtained by surgical resection. mRNA expression levels in healthy colon tissue of the eicosanoid enzymes, cyclooxygenase 2 (COX2) and 5-lipoxygenase (5-LOX), were included as markers of cellular stress induced by inflammation, tissue damage and/or hypoxia [15-19]. In addition, mRNA expression levels of glucose transporter 1 (GLUT-1) and carbonic anhydrase IX (CAIX) were included as markers of hypoxia [20,21].

## Results

### Surgical resection induces hypoxic cellular stress in healthy colon tissue

**Figure 1:**
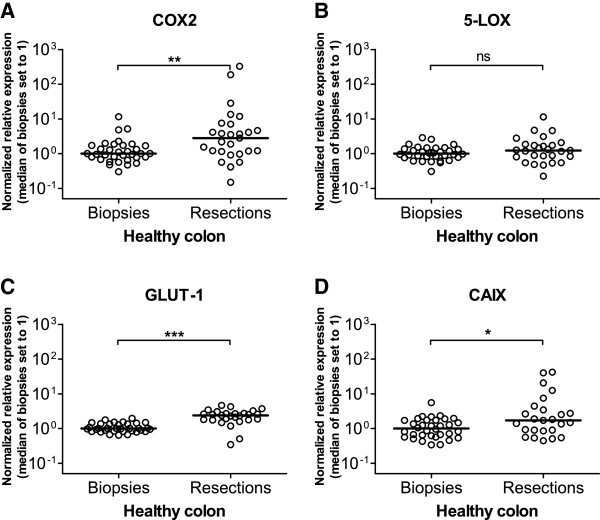
**Effect of sampling method on the expression of inflammatory and hypoxic stress responsive genes in healthy colon tissue samples.** Relative mRNA expression levels of the inflammatory eicosanoid enzymes COX2 (**A**) and 5-LOX (**B**) and of the hypoxia markers GLUT-1 (**C**) and CAIX (**D**) are shown for healthy colon biopsies and healthy colon resection samples. Expression levels were normalized against reference genes TBP and SDHA and were scaled against the median of the biopsy samples (median set to 1). Expression data are depicted as scatter plots of the values obtained for each individual sample. The horizontal line represents the median; ns: not significant; *: p < 0.05; **: p < 0.01; ***: p < 0.001 with Mann–Whitney *U* Test.

To examine to what extent the sampling procedure (biopsy *versus* surgical resection) may affect the overall condition of the sampled tissue, we analyzed the mRNA expression of COX2 and 5-LOX in samples of healthy colon tissue. As shown in Figure 1A, expression levels of *COX2*, an inflammation and hypoxia responsive gene used here as a biomarker of cellular stress, were significantly higher in resections than in biopsies. Also the expression levels of *GLUT-1* and *CAIX*, two hypoxia markers, were significantly increased in resected samples compared to biopsy samples (Figure 1C-D). Finally, the expression levels of *5-LOX*, included here as a control gene induced by cellular stress but insensitive to hypoxia, were identical in the two groups of samples (Figure 1B). Combined, these results indicate the induction by the surgical resection procedure of hypoxic cellular stress in the resected tissue.

### Surgical resection increases expression levels of VEGF family members in healthy colon tissue

**Figure 2:**
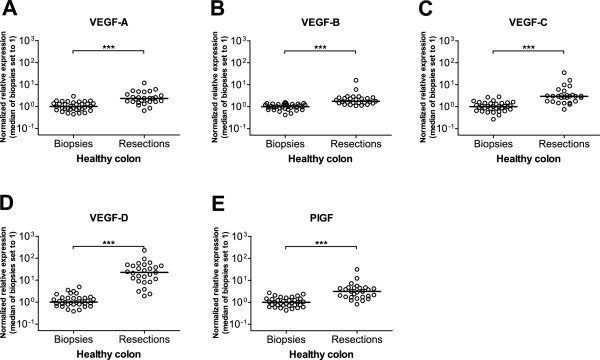
**Effect of sampling method on the expression of VEGF family members in healthy colon tissue samples.** Relative mRNA expression levels of VEGF-A (**A**), VEGF-B (**B**), VEGF-C (**C**), VEGF-D (**D**) and PlGF (**E**) are shown for healthy colon biopsies and healthy colon resection samples. Expression levels were normalized against reference genes TBP and SDHA and were scaled against the median of the biopsy samples (median set to 1). Expression data are depicted as scatter plots of the values obtained for each individual sample. The horizontal line represents the median; ***: p < 0.001 with Mann–Whitney *U* Test.

We next determined whether the occurrence of surgery-related hypoxic stress in resected healthy tissue samples was reflected in the expression levels of the individual VEGF family members. As shown in Figure 2, highly significant (p < 0.001) differences between resected and biopsy healthy colon samples were observed for all the VEGF family members. For these genes, the median expression levels were two- to three-fold (VEGF-A, -B, -C and PlGF) higher in resected than in biopsy samples, up to a striking 22-fold increase for VEGF-D.

### The sampling procedure affects the biomarker read-out of VEGF family members

**Figure 3:**
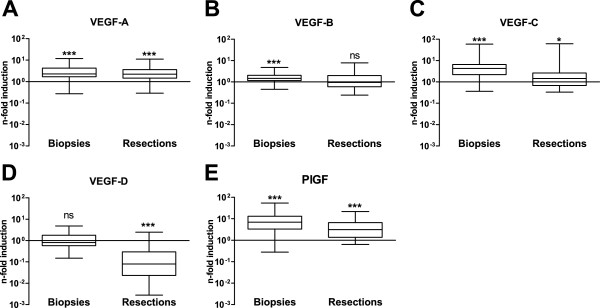
**Influence of sampling method on the biomarker read-out of VEGF family members.** n-Fold induction levels in carcinoma samples of VEGF-A (**A**), VEGF-B (**B**), VEGF-C (**C**), VEGF-D (**D**) and PlGF (**E**) are shown. The n-fold induction value represents the ratio of the expression value of the carcinoma sample against the expression value of the paired healthy sample. The box represents the median with interquartile range and the whiskers represent minimum and maximum ratios. ns: not significant; *:p < 0.05; ***: p < 0.001 with Wilcoxon signed-rank test.

We next assessed the extent to which the sampling-induced differences in VEGF gene expression observed in healthy tissue affected the magnitude of the difference between healthy and carcinoma tissue. To that end, we compared VEGF gene induction in colon carcinoma to matched healthy tissue samples obtained by biopsy or by surgical resection. Expression levels of *VEGF-A* were significantly induced in carcinoma tissues towards healthy tissues independent of the sampling method (Figure 3A). However, for the other VEGF family members, the magnitude of the difference between healthy and carcinoma tissue in resection samples was affected by the increment of expression in healthy tissue caused by the surgical sampling procedure. For *VEGF-B*, *VEGF-C* and *PlGF*, this resulted in reduced expression differences between healthy and carcinoma tissue in resected samples (Figure 3B, C and E). The consequences are most pronounced for VEGF-B that albeit significantly induced in biopsy carcinoma samples, no longer showed significance in carcinoma samples obtained by surgical resection (Figure 3B). A similar sampling procedure induced turnaround of biomarker value is observed for VEGF-D, although in an opposite direction. Here, the pronounced increase in the expression of *VEGF-D* in healthy resected tissue as opposed to the near absence of such an increase in carcinoma tissue resulted in a highly significant underexpression of *VEGF-D* in carcinoma resection samples (Figure 3D). On the contrary, in biopsy samples no difference in *VEGF-D* expression between healthy colon and colon carcinoma samples was observed.

### Cancer biomarker accuracy of VEGF family members

**Figure 4:**
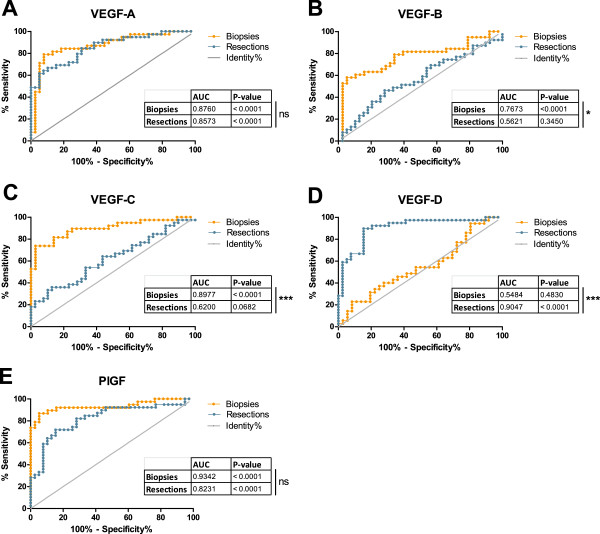
**ROC-analysis of the biomarker accuracy of VEGF family members for biopsy and resection samples.** ROC-curves of VEGF-A (**A**), VEGF-B (**B**), VEGF-C (**C**), VEGF-D (**D**) and PlGF (**E**) are shown for biopsy and resection samples. The ROC-curves represent the sensitivity and specificity of the individual VEGF family members as colon carcinoma biomarkers. The insert gives the area under the curve (AUC), which quantifies the ability of the marker to distinguish between healthy colon and colon carcinoma. The accompanying p-value tests the null hypothesis, namely, that the AUC equals 0.50 and thus the biomarker is incompetent. The identity-line (Identity%) represents the null hypothesis. *: p < 0.05; ***: p < 0.001 calculated with the method of DeLong et al. [35].

Receiver-operating characteristics (ROC) analysis is commonly used to assess the reliability and accuracy of potential biomarkers. ROC-based assessment of the individual VEGF family members as biomarkers for colon cancer identified overexpression of PlGF (AUC 0.9342) as the most effective mRNA-marker for samples obtained by biopsy with VEGF-A (AUC 0.8760) and VEGF-C AUC 0.8977) following as close seconds (Figure 4). This ranking however changes dramatically when considering samples obtained by resection. Here, underexpression of VEGF-D emerges as the most potent biomarker with an AUC of 0.9047 (p < 0.0001) and a ROC-curve significantly different (p < 0.0001) from the biopsy curve (Figure 4D). Overexpression of VEGF-A (AUC 0.8573) now precedes PlGF (AUC 0.8231), VEGF-C (AUC 0.6200) and especially VEGF-B (AUC 0.5621) shows strongly reduced accuracy as colon cancer mRNA-marker (Figure 4).

## Discussion

Biomarker expression profiles have become a valuable tool in diagnostic research, patient management and cancer therapy. We explored the influence of different sampling methods on the expression of VEGF family biomarkers in colon cancer. Samples obtained by either biopsy or surgical resection were compared for the differential expression of *VEGF-A*, *VEGF-B*, *VEGF-C*, *VEGF-D* and *PlGF*. To examine the occurrence of cellular stress caused by the sampling procedure, the expression levels of the eicosanoid enzymes *COX2* and *5-LOX* were quantified in healthy colon tissue. COX2 is a key inflammatory enzyme, and its expression is strongly induced by NF-κB and HIF-1 transcription factors in response to inflammatory insults and hypoxic growth conditions, respectively [19,22-26]. In contrast, expression of 5-LOX is largely insensitive to hypoxia but is similarly induced by various inflammatory insults [19,27,28]. Strikingly, we observed a pronounced expression increment of *COX2* in healthy colon resection samples relative to healthy biopsy samples. This was not the case for *5-LOX*. This differential expression pattern of *COX2* as opposed to *5-LOX* indicates that considerably more hypoxic stress may be present in resection samples than in samples obtained by biopsy. The presence of hypoxia in resection samples was further substantiated by the significantly increased expression in resection samples of the hypoxia markers GLUT-1 and CAIX. There is a large difference in the time needed to obtain samples by the two procedures. Whereas the collection of colon biopsies requires only minutes, surgical resection takes 30 to 90 minutes, during which the colon is clamped off. This cuts off blood circulation and oxygen delivery and could cause hypoxia in the clamped colon. The observed increment in COX2, GLUT-1 and CAIX mRNA levels in healthy colon tissue resections might therefore be a direct consequence of the clamping of part of the colon inducing a hypoxic stress signal.

VEGF-A is readily induced by COX2 derived prostaglandins such as PGE_2_[29,30]. Concomitant with the clear induction of COX2 mRNA in resected healthy tissue, *VEGF-A* expression levels were increased in healthy tissue resections. However, besides VEGF-A also other VEGF family members showed significant mRNA expression increments in resected healthy tissue ranging from 2–3 fold (VEGF-B, VEGF-C, PlGF) up to 22-fold (VEGF-D). Two recent reports described the induction during hypoxia of these VEGF family members in lung and lymphatic endothelial cells [31,32]. It is therefore likely that the combined action of clamping-induced hypoxia and COX2 derived prostaglandins are at the basis of the increased mRNA expression of VEGF-A as well as of the other VEGF family members we observed in resected healthy colon samples.

A determining factor in defining a biomarker is its accuracy in differentiating a healthy from a diseased state. Therefore, we assessed the ability of the individual VEGF members to discriminate between healthy and cancerous colon tissue and the influence of the sampling method on this ability. Although the cohort size (n=77) so far is rather limited, VEGF-A and PlGF emerged as potential mRNA-markers discriminating with relatively high accuracy between healthy and carcinoma tissue in samples obtained by biopsy or by surgical resection. Our results confirm previous studies reporting significantly increased expression levels of *VEGF-A* in colon carcinoma samples compared to healthy tissue [5-9]. The same conclusion holds true for PlGF. Of all VEGF family members, PlGF emerged from our ROC-analysis as the most accurate biomarker in both the sampling methods and was even more accurate than VEGF-A in biopsies. It is therefore remarkable that PlGF has received less attention than other VEGF members in colon carcinoma. Wei and colleagues studied resection samples from colorectal carcinoma patients and also documented increased PlGF mRNA expression levels and their association with reduced survival [9]. A similar result was obtained for both PlGF isoforms, PlGF-1 and PlGF-2, by Escudero-Esparza and colleagues [33]. Our observations further confirm these findings.

For VEGF-B, VEGF-C and VEGF-D we observed a significant impact of the sampling procedure on the mRNA expression levels in healthy *versus* colon carcinoma tissues. Table 1 compares our observations with previously reported data taking into account the reported sampling method but also other potential confounding factors such as the inclusion or not of rectal samples.

**Table 1:** Comparison of expression data for VEGF-B, VEGF-C and VEGF-D with previously published reports

**VEGF family member**	**Own data**		**Published data**			
	**Δ expression**		**Δ expression**	**Sampling method**	**Other confounders**	**Refs**
	**Resection**	**Biopsy**				
**VEGF-B**	=	↗ ***	=	Resection	-	[5]
			=	Resection	Rectal incl.	[7]
			=	Resection	Rectal incl.	[8]
**VEGF-C**	↗ *	↗ ***	=	Resection	-	[5]
			↗	n.s.	Rectal incl.	[6]
			↗	Resection	Rectal incl.	[7]
			=	Resection	Rectal incl.	[8]
			=	Biopsy	Rectal incl.	[14]
**VEGF-D**	↘ ***	=	↘	n.s.	Rectal incl.	[6]
			↘	Resection	Rectal incl.	[7]
			↘	Resection	Rectal incl.	[8]
			↘	Biopsy	Rectal incl.	[14]

Δ expression: differential expression in carcinoma samples compared to healthy tissue samples.

↗: significantly increased.

↘: significantly decreased.

=: no significance.

n.s.: not specified.

Rectal incl.: rectal samples included in the analysis.

*: p<0.05 with Wilcoxon signed rank test.

***: p<0.001 with Wilcoxon signed rank test.

Previous studies did not reveal overexpression of *VEGF-B* in colon carcinoma (Table 1). Also we did not observe increased VEGF-B mRNA levels in samples obtained by surgical resection. However, this lack of overexpression appears to be a consequence of the surgical sampling method rather than a characteristic intrinsic to colon carcinoma. This conclusion is based on the pronounced expression increment we observed in carcinoma tissue obtained by colonoscopic biopsy. These opposite results clearly identify the strong impact of the sampling procedure on VEGF-B mRNA-levels and challenge the conclusions of previous studies using samples obtained by surgical resection [5,7,8]. VEGF-C resembles VEGF-B in the impact of the clinical sampling method, showing a weak overexpression in resections as opposed to a pronounced, highly significant overexpression in biopsies (Table 1). Two out of five previously published reports similarly documented increased *VEGF-C* expression levels. Other reports using either biopsy or resected material failed however to detect significant changes. These conflicting data may be due to confounding factors other than the sampling method, namely the inclusion of rectal samples in these studies. Because radiotherapy prior to surgery is standard procedure in rectal cancer, we excluded such patients from our study. Finally, also VEGF-D shows a strong impact of the sampling method on its differential mRNA expression (Table 1). Here however, resected tissue samples show a pronounced underexpression as opposed to the absence of a differential expression in biopsy samples. This surgery-created signature again emphasizes the importance of taking into account the clinical procedure used for colon tissue sampling when performing colon cancer expression studies.

Our study included a total of 77 patients. Though this is a large cohort, clearly it is not large enough to exclude biases due to type I error. To detect type I errors, we statistically analyzed the likelihood that group-related disparities in gender, tumor grade, sample location and age confounded the conclusions of our study. As shown in the supplementary data (Additional file 1: Tables S1, Additional file 2: Table S2, Additional file 3: Table S3, Additional file 4: Table S4 and Additional file 5: Table S5), we did not detect specific biases that could contribute to the observed differential gene expression patterns. Yet, expansion of this study to a larger patient cohort may help to further corroborate our findings of direct relevance for colon cancer diagnosis and basic research.

## Conclusions

Our comparative gene mRNA expression analysis of healthy and carcinoma colon tissue shows that the sampling procedure - surgical resection *versus* colonoscopic biopsy - has an important impact on the read-out of VEGF family members as potential colon cancer mRNA-markers. The sampling-induced modulation of *VEGF* gene expression profiles could be related to cellular stress caused by hypoxia elicited in resected tissue samples by clamping of blood vessels during surgery. The higher sensitivity of healthy tissue to surgery-induced cellular stress compared to the relative insensitivity of carcinoma tissue affected to different degrees the reliability of individual VEGF-members as mRNA-markers for colon carcinoma. Therefore, samples obtained by biopsy provide a more reliable VEGF mRNA-marker read-out than samples obtained by surgical resection.

## Methods

### Biological samples

Samples were obtained from primary colon carcinomas either by biopsy (n=38) or by surgical resection (n = 39) at the Ghent University Hospital. Carcinomas were sampled in the infiltrating area of the growth, avoiding the necrotic center. Histopathological examination confirmed the carcinoma state of the tissue. From each patient, a corresponding healthy colon mucosa sample was taken from the same colon segment. None of the patients had received chemo- or radiotherapy before surgery or colonoscopy. Immediately after isolation, the biopsies were placed in RNA*later*® Solution (Ambion/Applied Biosystems, Foster City, CA). Surgical resection samples were also placed in RNA*later*® Solution at the end of the surgical procedure and after an initial examination by the pathologist. All samples were kept at −80°C until RNA extraction. The clinicopathological features of the patients are summarized in Table 2. All tissues were obtained following informed consent of the patients and approval of the study by the Ethics Committee of the Ghent University Hospital.

**Table 2:** Clinicopathological features of the colon carcinoma patients

**Variable**	**Number of patients**	
	**Biopsy**	**Resection**
Sex		
Male	22	25
Female	16	14
Age at diagnosis		
Median age (range, years)	70 (39–85)	67 (39–84)
Site of tumor		
Sigmoid	20	14
Colon descendens	2	2
Colon transversum	1	4
Hepatic flexure	1	3
Colon ascendens	5	5
Caecum and valve of Bauhin	8	7
Not specified	1	4
Tumor grade		
Low	4	5
Moderate	18	24
High	9	7
Unknown	7	3
Dukes classification		
Dukes’ A	3	8
Dukes’ B	15	12
Dukes’ C	6	12
Dukes’ D	13	5
Unknown	1	2
T category		
T1-T2	3	8
T3-T4	26	29
Tx	9	2
Lymphatic spread		
N0	18	21
N+	10	16
Nx	10	2
Metastasis		
M0	20	32
M+	13	5
Mx	5	2

### RNA extraction, RNA quality control and cDNA synthesis

Total RNA was extracted with the RNeasy Plus mini kit (Qiagen, Hilden, Germany) according to the manufacturer’s instructions. This kit contains a gDNA-elimination step to avoid gDNA contamination. After extraction, RNA quality and integrity was verified using an RNA 6000 Nano Chip Kit on the Agilent 2100 Bioanalyzer (Agilent Technologies, Santa Clara, CA, USA). Only samples with adequate quality and integrity (77/80) were used for the RT-qPCR analysis. cDNA was synthesized from 1 μg of total RNA using Superscript® II reverse transcriptase (Invitrogen, Merelbeke, Belgium) according to the manufacturer’s instructions.

### RT-qPCR

Real-time quantitative PCR (RT-qPCR) was performed using the LC 480 Sybr Green I master kit on a LightCycler® 480 Real-Time PCR system (both from Roche Applied Science, Penzberg, Germany). Primers were designed using PrimerSelect (DNASTAR, Madison, USA) and purchased from Invitrogen. The sequences of the forward and reverse primers were as follows: VEGF-A 5’-TGAGTTGCCCAGGAGACCAC-3’ and 5’-GAAGGGGAGCAGGAAGAGGAT-3’; VEGF-B 5’-CCGGAAGCTGCGAAGGTGACA-3’ and 5’-GGGAGACAAGGGATGGCAGAAGAG-3’; VEGF-C 5’-CACGGCTTATGCAAGCAAAGA-3’ and 5’-TCCTTTCCTTAGCTGACACTTGT-3’; VEGF-D 5’-GCAGCCCTAGAGAAACGTG-3’ and 5’-AGGTGCTGGTGTTCATACAGAT-3’; PlGF 5’-TGCGGCGATGAGAATCTGC-3’ and 5’-AGCGAACGTGCTGAGAGAAC-3’; COX2 5’-TTGCTGGAACATGGAATTACC-3’ and 5’-TGCCTGCTCTGGTCAATG-3’; 5-LOX 5’-TGGCGCGGTGGATTCATAC-3’ and 5’-CAGGGGAACTCGATGTAGTCC-3’; GLUT-1 5’-CTTGTGTGGCCTTCTTTGAAGT-3’ and 5’-CCACACAGTTGCTCCACAT-3’; CAIX 5’-GGAAGGCTCAGAGACTCA-3’ and 5’- CTTAGCACTCAGCATCAC-3’. All samples were assayed in triplicate. Relative expression values were calculated using the 2(−delta delta C(T)) method and were normalized against reference genes: tata-binding protein (*TBP*) and succinate dehydrogenase complex subunit A (*SDHA*) (primers: TBP 5’-CGGCTGTTTAACTTCGCTTC-3’ and 5'-CACACGCCAAGAAACAGTGA-3’; SDHA 5’-TGGGAACAAGAGGGCATCTG-3’ and 5’-CCACCACTGCATCAAATTCATG-3’). In these calculations we took into account the PCR efficiency of the individual PCR reactions, calculated on the basis of linear regression as described in Ruijter et al. [34]. For the comparison between healthy colon biopsies and resections, the normalized relative expression values were scaled against the median of the healthy biopsies (median of biopsies set to 1). The specificity of amplification was confirmed by evaluation of the melting curves.

### Statistical analysis

Statistical analysis was performed using the GraphPad Prism® software (GraphPad Software Inc., La Jolla, California, USA). Statistical significance of comparisons between two independent groups was determined with the two-tailed Mann–Whitney *U* test. The comparison between paired samples was performed with the Wilcoxon signed-rank test. The accuracy of the markers was determined with receiver operator characteristic curves (ROC). The statistical significance of the difference between two areas under the ROC curves was calculated by the method of DeLong et al. and performed with MedCalc® software (MedCalc Software, Mariakerke, Belgium) [35]. Significant p-values were ranked as p < 0.05 (*), p < 0.01 (**) and p < 0.001 (***).

## Abbreviations

5-LOX: 5-lipoxygenase; AUC: Area under the curve; CAIX: Carbonic anhydrase IX; COX2: Cyclooxygenase 2; GLUT-1: Glucose transporter 1; HIF-1: Hypoxia inducible factor 1; NFκB: Nuclear factor kappa B; PlGF: Placental growth factor; ROC: Receiver operator characteristic; RT-qPCR: Real-time quantitative PCR; SDHA: Succinate dehydrogenase complex subunit A; TBP: Tata-binding protein; VEGF: Vascular endothelial growth factor.

## Competing interests

The authors declare that they have no competing interests.

## Authors’ contributions

SP conceived and initiated this project, performed RNA extractions, RT-qPCR assays and data analysis, and wrote the manuscript. NVD participated in the design and coordination of the study, coordinated the acquisition of patient data and clinical samples, and revised the manuscript. BDC contributed to the experimental design and the data analysis and revised the manuscript. PP and WC provided clinical samples and clinicopathological data and revised the manuscript. MP conceived the study, participated in the design, and revised the manuscript. JG conceived the study, and participated in its design and coordination and helped to draft the manuscript. All authors read and approved the final manuscript.

## Pre-publication history

The pre-publication history for this paper can be accessed here:

http://www.biomedcentral.com/1471-2407/12/515/prepub

## Supplementary Material

Additional file 1 **Table S1.** Comparison of expression levels in male versus female patients with Mann-Whitney test. *: p < 0.05. Click here for file

Additional file 2 **Table S2.** Comparison of expression levels in colon carcinoma with tumor grade low versus moderate versus high with Kruskal Wallis test. *: p < 0.05. Click here for file

Additional file 3 **Table S3.** Comparison of expression levels in colon carcinoma with Dukes classification A versus B versus C versus D with Kruskal Wallis test. *: p < 0.05. Click here for file

Additional file 4 **Table S4.** Comparison of expression levels in healthy colon and colon carcinoma samples from different tumor sites (caecum and Valve of Bauhin versus colon ascendens, transversum, descendens and hepatic flexure versus sigmoid) with Kruskal Wallis test. *: p < 0.05; **:p < 0.01. n/a: not applicable. Click here for file

Additional file 5 **Table S5.** Comparison of expression levels in patients younger than 70 years or of 70 years versus patients older than 70 with Mann-Whitney test. Click here for file
