# Supplementary material for: Clinical procedure for colon carcinoma tissue sampling directly affects the cancer marker-capacity of VEGF family members
Source: BMC Cancer. 2012 Nov 13;12:515. doi: 10.1186/1471-2407-12-515 (PMC3534223; doi:10.1186/1471-2407-12-515)
Supplement: Additional file 1 — Table S1. Comparison of expression levels in male versus female patients with Mann-Whitney test. *: p < 0.05. [file 1471-2407-12-515-S1.docx]

| **Gene** | **Biopsies** | | | | **Resections** | | | |
| --- | --- | --- | --- | --- | --- | --- | --- | --- |
|  | Healthy colon | | Colon carcinoma | | Healthy colon | | Colon carcinoma | |
|  | p-Value | Sign diff? ^1^ | p-Value | Sign diff? ^1^ | p-Value | Sign diff? ^1^ | p-Value | Sign diff? ^1^ |
| **COX2** | 0.6119 | no | - | - | 0.4253 | no | - | - |
| **5-LOX** | 0.9860 | no | - | - | 0.3413 | no | - | - |
| **GLUT-1** | 0.9025 | no | - | - | 0.2268 | no | - | - |
| **CAIX** | 0.6618 | no | - | - | 0.3159 | no | - | - |
| **VEGF-A** | 0.1008 | no | 0.8708 | no | 0.9184 | no | 0.9417 | no |
| **VEGF-B** | 0.0948 | no | 0.5643 | no | 0.6290 | no | 0.4732 | no |
| **VEGF-C** | 0.1315 | no | 0.3831 | no | 0.0868 | no | 0.8721 | no |
| **VEGF-D** | 0.0426 | * | 0.0765 | no | 0.7363 | no | 0.4642 | no |
| **PlGF** | 0.2312 | no | 0.9176 | no | 0.8952 | no | 0.9184 | no |
| ^1^ Sign diff?: Significant difference between samples from male or female patients? | | | | | | | | |

Table S1: Comparison of expression levels in male versus female patients with Mann-Whitney test. *: p<0.05
