# Supplementary material for: Clinical procedure for colon carcinoma tissue sampling directly affects the cancer marker-capacity of VEGF family members
Source: BMC Cancer. 2012 Nov 13;12:515. doi: 10.1186/1471-2407-12-515 (PMC3534223; doi:10.1186/1471-2407-12-515)
Supplement: Additional file 2 — Table S2. Comparison of expression levels in colon carcinoma with tumor grade low versus moderate versus high with Kruskal Wallis test. *: p < 0.05. [file 1471-2407-12-515-S2.docx]

| **Gene** | **Biopsies** | | | | **Resections** | | | |
| --- | --- | --- | --- | --- | --- | --- | --- | --- |
|  | Healthy colon | | Colon carcinoma | | Healthy colon | | Colon carcinoma | |
|  | p-Value | Sign diff? ^1^ | p-Value | Sign diff? ^1^ | p-Value | Sign diff? ^1^ | p-Value | Sign diff? ^1^ |
| **COX2** | 0.0883 | no | - | - | 0.0397 | * | - | - |
| **5-LOX** | 0.4667 | no | - | - | 0.1646 | no | - | - |
| **GLUT-1** | 0.5942 | no | - | - | 0.7709 | no | - | - |
| **CAIX** | 0.3980 | no | - | - | 0.6819 | no | - | - |
| **VEGF-A** | 0.5775 | no | 0.3662 | no | 0.6067 | no | 0.2818 | no |
| **VEGF-B** | 0.8955 | no | 0.8589 | no | 0.6085 | no | 0.6771 | no |
| **VEGF-C** | 0.9849 | no | 0.5805 | no | 0.1073 | no | 0.0321 | * |
| **VEGF-D** | 0.4946 | no | 0.6100 | no | 0.6195 | no | 0.1850 | no |
| **PlGF** | 0.0394 | * | 0.9056 | no | 0.2856 | no | 0.8994 | no |
| ^1^ Sign diff?: Significant difference between samples from different tumor grade? | | | | | | | | |

Table S2: Comparison of expression levels in colon carcinoma with tumor grade low versus moderate versus high with Kruskal Wallis test. *: p<0.05
