# Supplementary material for: Clinical procedure for colon carcinoma tissue sampling directly affects the cancer marker-capacity of VEGF family members
Source: BMC Cancer. 2012 Nov 13;12:515. doi: 10.1186/1471-2407-12-515 (PMC3534223; doi:10.1186/1471-2407-12-515)
Supplement: Additional file 3 — Table S3. Comparison of expression levels in colon carcinoma with Dukes classification A versus B versus C versus D with Kruskal Wallis test. *: p < 0.05. [file 1471-2407-12-515-S3.docx]

| **Gene** | **Biopsies** | | | | **Resections** | | | |
| --- | --- | --- | --- | --- | --- | --- | --- | --- |
|  | Healthy colon | | Colon carcinoma | | Healthy colon | | Colon carcinoma | |
|  | p-Value | Sign diff?^1^ | p-Value | Sign diff? ^1^ | p-Value | Sign diff? ^1^ | p-Value | Sign diff? ^1^ |
| **COX2** | 0.6734 | no | - | - | 0.6011 | no | - | - |
| **5-LOX** | 0.3685 | no | - | - | 0.0613 | no | - | - |
| **GLUT-1** | 0.3680 | no | - | - | 0.1770 | no | - | - |
| **CAIX** | 0.6838 | no | - | - | 0.6578 | no | - | - |
| **VEGF-A** | 0.6942 | no | 0.7199 | no | 0.0800 | no | 0.3722 | no |
| **VEGF-B** | 0.7275 | no | 0.6361 | no | 0.0337 | * | 0.1204 | no |
| **VEGF-C** | 0.8051 | no | 0.5012 | no | 0.1017 | no | 0.1844 | no |
| **VEGF-D** | 0.4686 | no | 0.7552 | no | 0.1827 | no | 0.6879 | no |
| **PlGF** | 0.7782 | no | 0.7614 | no | 0.0993 | no | 0.1650 | no |
| ^1^ Sign diff?: Significant difference between samples with different Dukes classification? | | | | | | | | |

Table S3: Comparison of expression levels in colon carcinoma with Dukes classification A versus B versus C versus D with Kruskal Wallis test. *: p<0.05
