# Supplementary material for: Clinical procedure for colon carcinoma tissue sampling directly affects the cancer marker-capacity of VEGF family members
Source: BMC Cancer. 2012 Nov 13;12:515. doi: 10.1186/1471-2407-12-515 (PMC3534223; doi:10.1186/1471-2407-12-515)
Supplement: Additional file 4 — Table S4. Comparison of expression levels in healthy colon and colon carcinoma samples from different tumor sites (caecum and Valve of Bauhin versus colon ascendens, transversum, descendens and hepatic flexure versus sigmoid) with Kruskal Wallis test. *: p < 0.05; **:p < 0.01. n/a: not applicable. [file 1471-2407-12-515-S4.docx]

| **Gene** | **Biopsies** | | | | **Resections** | | | |
| --- | --- | --- | --- | --- | --- | --- | --- | --- |
|  | Healthy colon | | Colon carcinoma | | Healthy colon | | Colon carcinoma | |
|  | p-Value | Sign diff?^1^ | p-Value | Sign diff? ^1^ | p-Value | Sign diff? ^1^ | p-Value | Sign diff? ^1^ |
| **COX2** | n/a | n/a | - | - | n/a | n/a | - | - |
| **5-LOX** | n/a | n/a | - | - | n/a | n/a | - | - |
| **GLUT-1** | n/a | n/a | - | - | n/a | n/a | - | - |
| **CAIX** | n/a | n/a | - | - | n/a | n/a | - | - |
| **VEGF-A** | n/a | n/a | 0.0671 | no | n/a | n/a | 0.7597 | no |
| **VEGF-B** | n/a | n/a | 0.5926 | no | n/a | n/a | 0.7546 | no |
| **VEGF-C** | n/a | n/a | 0.2775 | no | n/a | n/a | 0.7456 | no |
| **VEGF-D** | n/a | n/a | 0.8341 | no | n/a | n/a | 0.7937 | no |
| **PlGF** | n/a | n/a | 0.5477 | no | n/a | n/a | 0.9067 | no |
| ^1^ Sign diff?: Significant difference between samples from different tumor sites? | | | | | | | | |

Table S4: Comparison of expression levels in healthy colon and colon carcinoma samples from different tumor sites (caecum and Valve of Bauhin versus colon ascendens, transversum, descendens and hepatic flexure versus sigmoid) with Kruskal Wallis test. *: p<0.05; **:p<0.01. n/a: not applicable.
