# Supplementary material for: Clinical procedure for colon carcinoma tissue sampling directly affects the cancer marker-capacity of VEGF family members
Source: BMC Cancer. 2012 Nov 13;12:515. doi: 10.1186/1471-2407-12-515 (PMC3534223; doi:10.1186/1471-2407-12-515)
Supplement: Additional file 5 — Table S5. Comparison of expression levels in patients younger than 70 years or of 70 years versus patients older than 70 with Mann-Whitney test. [file 1471-2407-12-515-S5.docx]

| **Gene** | **Biopsies** | | | | **Resections** | | | |
| --- | --- | --- | --- | --- | --- | --- | --- | --- |
|  | Healthy colon | | Colon carcinoma | | Healthy colon | | Colon carcinoma | |
|  | p-Value | Sign diff? ^1^ | p-Value | Sign diff? ^1^ | p-Value | Sign diff? ^1^ | p-Value | Sign diff? ^1^ |
| **COX2** | 0.1729 | no | - | - | 0.7113 | no | - | - |
| **5-LOX** | 0.3979 | no | - | - | 0.9803 | no | - | - |
| **GLUT-1** | 0.5928 | no | - | - | 0.0887 | no | - | - |
| **CAIX** | 0.0136 | * | - | - | 0.7861 | no | - | - |
| **VEGF-A** | 0.7927 | no | 0.6827 | no | 0.1227 | no | 0.9210 | no |
| **VEGF-B** | 0.4835 | no | 0.9767 | no | 0.0490 | * | 0.3013 | no |
| **VEGF-C** | 0.2674 | no | 0.6197 | no | 0.0490 | * | 0.2514 | no |
| **VEGF-D** | 0.2540 | no | 0.3818 | no | 0.1127 | no | 0.1975 | no |
| **PlGF** | 0.7703 | no | 0.6404 | no | 0.6403 | no | 0.1879 | no |
| ^1^ Sign diff?: Significant difference between samples from patients of ≤70 years or >70 years? | | | | | | | | |

Table S1: Comparison of expression levels in patients younger than 70 years or of 70 years versus patients older than 70 with Mann-Whitney test.
